# CropGS-Hub: a comprehensive database of genotype and phenotype resources for genomic prediction in major crops

**DOI:** 10.1093/nar/gkad1062

**Published:** 2023-11-24

**Authors:** Jiaxin Chen, Cong Tan, Min Zhu, Chenyang Zhang, Zhihan Wang, Xuemei Ni, Yanlin Liu, Tong Wei, XiaoFeng Wei, Xiaodong Fang, Yang Xu, Xuehui Huang, Jie Qiu, Huan Liu

**Affiliations:** Shanghai Key Laboratory of Plant Molecular Sciences, Shanghai Collaborative Innovation Center of Plant Germplasm Resources, College of Life Sciences, Shanghai Normal University, Shanghai, China; State Key Laboratory of Agricultural Genomics, Key Laboratory of Genomics, Ministry of Agriculture, BGI Research, Shenzhen 518083, China; BGI Research, Wuhan 430074, China; Shanghai Key Laboratory of Plant Molecular Sciences, Shanghai Collaborative Innovation Center of Plant Germplasm Resources, College of Life Sciences, Shanghai Normal University, Shanghai, China; State Key Laboratory of Agricultural Genomics, Key Laboratory of Genomics, Ministry of Agriculture, BGI Research, Shenzhen 518083, China; BGI Bioverse, Shenzhen 518083, China; Shanghai Key Laboratory of Plant Molecular Sciences, Shanghai Collaborative Innovation Center of Plant Germplasm Resources, College of Life Sciences, Shanghai Normal University, Shanghai, China; State Key Laboratory of Agricultural Genomics, Key Laboratory of Genomics, Ministry of Agriculture, BGI Research, Shenzhen 518083, China; BGI Bioverse, Shenzhen 518083, China; Shanghai Key Laboratory of Plant Molecular Sciences, Shanghai Collaborative Innovation Center of Plant Germplasm Resources, College of Life Sciences, Shanghai Normal University, Shanghai, China; State Key Laboratory of Agricultural Genomics, Key Laboratory of Genomics, Ministry of Agriculture, BGI Research, Shenzhen 518083, China; BGI Research, Wuhan 430074, China; China National GeneBank, BGI, Shenzhen 518120, China; State Key Laboratory of Agricultural Genomics, Key Laboratory of Genomics, Ministry of Agriculture, BGI Research, Shenzhen 518083, China; BGI Research, Sanya 572025, China; Agricultural College, Yangzhou University, Yangzhou 225009, China; Shanghai Key Laboratory of Plant Molecular Sciences, Shanghai Collaborative Innovation Center of Plant Germplasm Resources, College of Life Sciences, Shanghai Normal University, Shanghai, China; Shanghai Key Laboratory of Plant Molecular Sciences, Shanghai Collaborative Innovation Center of Plant Germplasm Resources, College of Life Sciences, Shanghai Normal University, Shanghai, China; State Key Laboratory of Agricultural Genomics, Key Laboratory of Genomics, Ministry of Agriculture, BGI Research, Shenzhen 518083, China; BGI Bioverse, Shenzhen 518083, China

## Abstract

The explosive amount of multi-omics data has brought a paradigm shift both in academic research and further application in life science. However, managing and reusing the growing resources of genomic and phenotype data points presents considerable challenges for the research community. There is an urgent need for an integrated database that combines genome-wide association studies (GWAS) with genomic selection (GS). Here, we present CropGS-Hub, a comprehensive database comprising genotype, phenotype, and GWAS signals, as well as a one-stop platform with built-in algorithms for genomic prediction and crossing design. This database encompasses a comprehensive collection of over 224 billion genotype data and 434 thousand phenotype data generated from >30 000 individuals in 14 representative populations belonging to 7 major crop species. Moreover, the platform implemented three complete functional genomic selection related modules including phenotype prediction, user model training and crossing design, as well as a fast SNP genotyper plugin-in called SNPGT specifically built for CropGS-Hub, aiming to assist crop scientists and breeders without necessitating coding skills. CropGS-Hub can be accessed at https://iagr.genomics.cn/CropGS/.

## Graphical Abstract

**Graphical Abstract:**
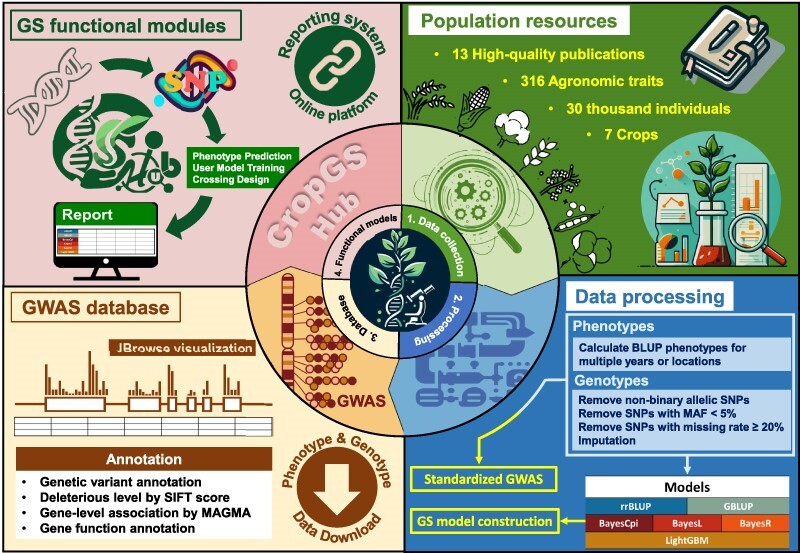

## Introduction

In the past few decades, an immense volume of genomic and phenotype data has been accumulated through crop population genomics studies. These datasets offer great potential in deciphering the genetic mechanisms behind crop development and facilitating genomically designed breeding aided by multi-omics data and artificial intelligence algorithms. However, the lack of relevant, well-curated, and integrated databases, as well as code-free analysis applications, presents significant challenges for effectively reusing these valuable resources.

To address these challenges, numerous efforts have been made by research communities to promote the free access and efficient reuse of data resources, particularly regarding whole genome associations. Several databases and webservers have been established, such as NHGRI-EBI GWAS Catalog (1), GWAS Central (2), GWASdb (3), GWAS Atlas (4) and easyGWAS (5). Among these, the GWAS Catalog, GWASdb and GWAS Central were created to curate published genome-wide association studies in humans and model animals, aiming to identify causal variants and understand disease mechanisms for the development of novel therapies (1–3). GWAS Atlas serves as a comprehensive GWAS database covering a wide range of species, including both plants and animals (4). On the other hand, easyGWAS offers a powerful, species-independent online resource for computing, storing, sharing, annotating, and comparing GWAS (5). It provides users with conveniences to replicate GWAS signals and enhance the detection power in GWAS studies. These initiatives aim at facilitating access to valuable genomic and phenotype data and promoting further research in the field of crop population genomics.

While several informative crop GWAS databases contain associations between phenotypes and genotypes, only a few databases fully leverage phenotype and genotype resources to construct and host genome prediction models for agronomic traits. Genomic prediction (GP) refers to the use of advanced statistical, machine-learning or deep-learning models and genetic markers to predict the phenotypic traits or performance of the target individuals. GP has proven useful in diagnosing genetic diseases in human health and accelerating the rate of genetic gain in plant and animal breeding (6,7). This enables breeders to prioritize plants with the highest potential for preferred traits at early stages, accelerating the breeding process and improving the efficiency of trait selection in plant breeding programs. The availability of high-quality training population data resources is crucial for the accuracy of genomic prediction and precise breeding in plants. Unfortunately, there exists limited number of databases or online applications capable of the practical use of genomic prediction in crops.

**Figure 1.**
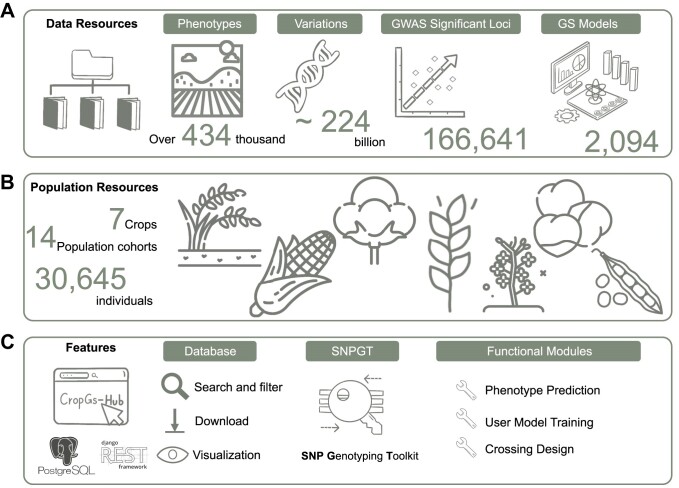
Overview of data resources and features of CropGS-Hub. (**A**) The number of phenotypes, genotypes, GWAS associations and GS models in CropGS-Hub. (**B**) Information of species and individual number for the cohorts. (**C**) Summary of features and functional modules in CropGS-Hub.

To address these gaps, we have created CropGS-Hub (URL: https://iagr.genomics.cn/CropGS), a comprehensive database that integrates genotype, phenotype, and GWAS resources, as well as a one-stop platform incorporating algorithms for genomic prediction and crossing design (Figure 1). Within this database, we have systematically curated extensive datasets comprising over 224 billion genotype data points and 434 000 phenotype data points from >30 000 individuals across 14 populations of 7 major crop species. These resources, along with 166 641 GWAS associations, can be easily searched and freely downloaded. Additionally, variant annotations, including variant impact and deleterious levels, are provided to identify causal genes responsible for specific traits. CropGS-Hub also features three major online analysis modules related to genomic selection: ‘Phenotype Prediction’, ‘User Model Training’ and ‘Crossing Design’. Each trait is trained using six models, including traditional statistical methods and machine learning approaches. We have integrated these constructed genome prediction model resources, along with a fast SNP genotyping utility called SNPGT, into our database. CropGS-Hub empowers crop scientists and breeders to effortlessly perform genome selection, minimizing the need for extensive programming or command-line skills.

In conclusion, CropGS-Hub plays a pivotal role in accelerating genome-designed breeding and advancing modern breeding with precision and efficiency. By promoting the free sharing and efficient utilization of invaluable data resources for precise breeding in major crops, CropGS-Hub greatly benefits the research community and helps address challenges related to food insecurity.

## Materials and methods

### Collection of datasets

A total of 14 high-quality cohorts from 7 important crops, namely maize (*Zea mays*), rice (*Oryza sativa*), cotton (*Gossypium hirsutum*), millet (*Setaria italica*), chickpea (*Cicer arietinum*), rapeseed (*Brassica napus*), and soybean (*Glycine max*), were collected from 13 published papers (8–20). In Supplementary Table S1, we have listed the detailed resources, including phenotype data, genotype data, the list of germplasm resources, reference genome, and annotation files.

Among these cohorts, rice and maize have 3 and 6 cohorts, respectively. For rice, there are 2 inbred rice cohorts (8,9) and 1 hybrid rice cohort (10). The first two cohorts consist of inbred samples from different subgroups such as *indica*, *aus*, temperate *japonica*, tropical *japonica*, and *basmati*. The hybrid rice cohort includes samples from 3 hybridization groups: *indica* × *indica*, *japonica* × *japonica* and *indica* × *japonica*. Similarly, maize comprises 6 cohorts, with 2 consisting of inbred lines, one complete-diallel design plus Unbalanced Breeding-like Inter-Cross (CUBIC) population, one CUBIC derivative population, and two CUBIC derivative hybrid lines. The inbred lines consist of elite lines from China and the United States (11,12). The CUBIC lines are a genetic population derived from 24 Chinese elite inbred parental lines (13). The maize hybrid line cohort includes individuals crossed by 30 elite lines with CUBIC lines (14,15). The remaining 5 cohorts are from cotton (16), millet (17), chickpea (18), rapeseed (19) and soybean (20), with one cohort for each species.

### Data cleaning

Based on the sample classification mentioned in the 13 articles, samples of crop wild relatives were initially removed for downstream analyses. For traits with data collected from multiple years or locations, the Best Linear Unbiased Predictors (BLUP) values for each sample were calculated using the R package lme4 (21). Both the BLUP values and original phenotypes were used for subsequent GWAS analysis and GS model construction.

Multi-allelic SNPs and SNPs with a minor allele frequency (MAF) <0.05 were eliminated. Additionally, SNPs with a genotype missing ratio exceeding 0.2 were filtered out using Plink (v1.9) (22). Finally, missing genotypes were imputed using Beagle (v4.1) (23).

### Standardized GWAS and variant annotation

The phenotype and imputed genotype data mentioned earlier were used to perform GWAS using GEMMA (v0.98) (24). Pairwise kinship matrices were created using the centered genomic relationship matrix (GRM) algorithm in GEMMA, based on the quality control and imputation SNPs of each cohort. Univariate linear mixed models (LMM) in GEMMA were then employed to conduct association tests. To estimate the effective number of markers, the Genetic Type I error calculator (GEC) (v0.2) (25) was used. The genome-wide significance threshold for all traits was set at a uniform threshold of *P* = 1/the effective number of independent SNPs.

Potential variant annotation and effect were predicted by SnpEff v3.6 (26), and SIFT4G (27) were utilized to evaluate the effect and measure the conservation level. The protein sequences of each species were obtained using the gffread tool in cufflinks (v2.2.1) (28) based on the provided genome file. BLASTP (v2.6.0) was then employed to annotate the sequences using *Arabidopsis thaliana* protein sequences as references, with an *E*-value threshold of 1e-5. The gene-level association analysis was performed by the Multi-marker Analysis of GenoMic Annotation (MAGMA) (v1.10) (29).

### Evaluating the effect of SNP type on genomic prediction accuracy

Genome-wide SNPs were classified into five types: intergenic SNPs, intron SNPs, synonymous SNPs, tolerated SNPs (tSNP), and deleterious SNPs (dSNPs). The first three types could be determined using reference genome and annotation files based on SnpEff annotation, while the nonsynonymous SNPs could be further classified based on the SIFT score (dSNP: SIFT < 0.05; tSNP: SIFT ≥ 0.05).

As different SNP types have different numbers of markers, we down-sampled the number of each SNP type to 10 000. This down-sampling process was repeated 30 times for each of the five SNP types. For each set of SNPs, 10-fold cross-validation was performed 20 times. The Pearson correlation (*r*) between the predicted phenotype and the observed phenotype in the validation set was calculated for each 10-fold cross-validation as the prediction accuracy for the down-sampled SNP set. Among the 20 × 30 repetitions, the highest *r* value was chosen as the prediction accuracy for the specific SNP type.

### Genomic selection model construction

After performing quality control on the SNPs of each cohort, we selected the synonymous SNPs (ssSNPs) and conducted linkage disequilibrium (LD) pruning with Plink (v1.9) (22) using an *r*^2^ threshold of <0.5. The remaining variant loci (ssSNP_LD0.5) were used as genotypic loci for constructing genomic selection (GS) models. We employed six different GS algorithms, including traditional linear models such as rrBLUP (30), GBLUP (31), BayesCpi, BayesL, and BayesR (32), as well as the machine learning-based software LightGBM (33). For the five linear model-based methods, we calculated the correlation coefficient (*r*) obtained from 20 repetitions of 10-fold cross-validation, and selected the model with the highest *r* as the GS prediction model for the trait. For LightGBM, we trained our model using gradient boosting decision tree and regression models. The learning rate was set to 0.1, and we tuned four hyperparameters (number of leaves, maximum depth, minimum data in leaf, and feature fraction) using grid search to find the optimal values. The selection method for the best model for LightGBM was the same as that for the linear models.

### Implementation of SNPGT module for CropGS-Hub

To make genome selection more convenient for users to perform with their own sample data, we implemented a utility called SNPGT (https://iagr.genomics.cn/CropGS/#/snpgt) for the CropGS-Hub website (Supplementary Figure S1). This utility allows for rapid genotyping of target SNPs using local machine. Unlike the genome-wide SNP calling pipeline, we first extracted the tag sequences around the SNP sites (up and down 200 bp). The users’ sequencing reads were then imported and mapped to the tag sequences using Bowtie2 (34), which is designed to *in silico* collect reads useful for target site genotyping of specific cohort. Mappable reads were collected using Samtools v1.2 (35). These collected reads were then re-mapped to the entire reference genome using Bowtie2. For each sample, we used GATK v3.8 (36) to perform the genotyping for the target SNPs. We have developed a pipeline (LinSNPGT) for Linux-based users using common Python scripts. For Windows-system users, we have created a user-friendly interface (WinSNPGT) using PyWebIO. All Linux-based bioinformatic software and scripts have been packaged using Cygwin (37).

In order to evaluate the efficiency and accuracy of SNPGT, we used rice cohort GSTP008 (containing 6215 SNP sites) as an example. The simulated NGS data was generated using dwgsim (https://github.com/nh13/DWGSIM) based on the assembled rice genome of 9311, with sequencing depth of 1, 2, 5, 10, 15 and 20×. The simulated sequencing data of different depths were performed for mapping and genotyping of the 6215 SNP sites using both traditional SNP genotyping method and pipeline implemented in SNPGT (Supplementary Figure S1).

### CropGS-Hub implementation

CropGS-Hub was developed on a cloud server with elastic computing resources (with 32 CPUs, 64 GB RAM and 1000 Gb storage) using open-source frameworks and software including Python (v3.9.12)/Django (v3.2.5), web technologies (HTML, CSS), JavaScript/Vue, Nginx (v1.22.0), and Celery (v4.4.7). PostgreSQL (v14.4) and Redis (v7.0.4) applications were used for backend database management, while the built-in online pipelines were developed using shell scripting, Python (v3.7.12), Perl (v5.26.1) and R languages (v3.3.4).

### Development of mini-core collections for fast genotype imputation

When users’ sample genotypes are submitted to CropGS-Hub and integrated with the genotype matrix in the database for genomic selection, it is necessary to perform genotype imputation for their samples, as missing genotypes are not allowed for GS softwares. Because using all individuals as the imputation genetic background would be exhaustive and inefficient, we utilized an R package called ‘corehunter’ (38) to develop an independent mini-core collection based on the phenotype and genotype data. For each of the 12 cohorts with more than 500 samples, a cutoff ratio corresponding to the size of the parameter was set to select 500 samples as the output mini-core collection (Supplementary Figure S2). The imputation pipeline for the users’ samples were implemented to use Beagle v5.4 (39)

### Performing prediction cases

We acquired genome re-sequencing data for a total of 295 parents from Lv et al. (40), consisting of the male sterile line Quan9311A and another 294 3-line restorer lines (Supplementary Table S3). Subsequently, we obtained their genotypic data for SNP sites of GSTP007 utilizing SNPGT. After uploading the parental genotypes to the ‘Crossing Design’ module, GSTP007 was designated as the training set. Six models were employed to predict the phenotypes of two yield-related traits (‘Grain number per panicle BLUP’ and ‘Yield per plant BLUP’) for the hybrids resulting from crosses between Quan9311A and the 294 restorer lines.

We selected the flesh sweetness of watermelon as a case study to demonstrate the functionality of our ‘User Model Training’ feature in CropGS-Hub. The phenotype and genotype data for watermelon accessions were obtained from Guo *et al.* (41) (Supplementary Table S3). For our analysis, we randomly selected a set of 30 samples as a test population, while the remaining 229 samples were used for training using a 10-fold cross-validation approach. In the ‘User Model Training’ interface, the genotype and phenotype data of the 229 training samples were submitted. Additionally, the genotype data of the 30 test samples was also uploaded for phenotype prediction.

## Results

### Data collection, processing and database content

#### Content of genotype and phenotype data

**Figure 2.**
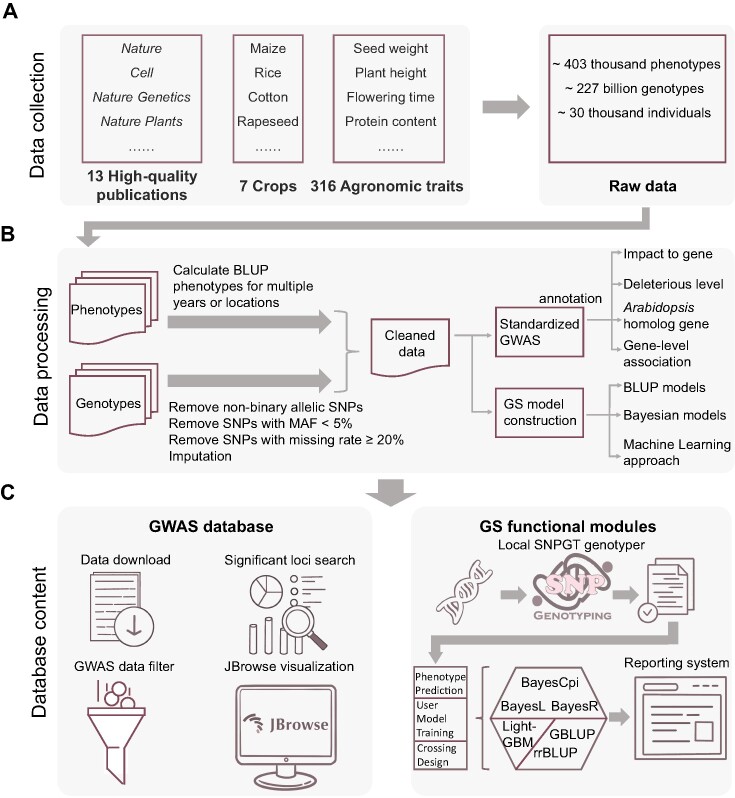
Summary of data collection, data processing, and database content of CropGS-Hub. (**A**) Process of cohort data collection. (**B**) Flowchart depicting the cleaning process of phenotypic and genotypic data, followed by subsequent analysis. (**C**) Overview of the database content, categorized into the GWAS database and the GS functional modules.

CropGS-Hub has curated a total of 14 cohorts covering 7 major crops, including maize, rice, cotton, foxtail millet, chickpea, rapeseed, and soybean (Figure 2A). The number of individuals in each cohort ranges from 350 to 8652, while the number of SNPs spans from 161 562 to 31 580 805, with a median number of 4.5 million (Supplementary Table S1). As for phenotypes, each cohort involves 3 to 111 traits, which contains a range of agronomic traits, such as flowering time-related traits (e.g. days to silking, days to anthesis), plant architecture (e.g. plant height, panicle length, leaf width), and yield components (e.g. grain yield per plant, seed weight) (Supplementary Table S2).

#### GWAS resources for 7 major crops

GWAS databases usually collect GWAS summary statistics from published journals. However, this approach can introduce bias due to variations in methodologies, parameters, and even threshold criteria, leading to inconsistent GWAS outcomes. Using a standardized GWAS approach and uniform significance threshold, a total number of 166 641 significant associated SNPs were detected and annotated with functional information, including gene impact, deleterious level, genetic effect and the Arabidopsis homolog of the affected gene (Figure 2B). These genetic resources can be easily searched and filtered in the ‘Datasets’ section of CropGS-Hub, and the JBrowse feature allows for dynamic visualization of GWAS results (Figure 2C).

#### Construction of GS functional modules

To explore the genetic relationships between genotype and phenotype datasets, we have designed GS functional modules for CropGS-Hub. Before constructing GS models, it is vital to determine which variant sites to include. We classified genomic SNPs into five types: intergenic SNPs, intronic SNPs, synonymous SNPs, tolerated non-synonymous SNPs, and deleterious SNPs. During benchmarking using three traits of three crops (maize, rice and soybean), we observed that prediction performance based on synonymous SNPs was better compared to the other four variant types (ranking first for 5 of 9 times, ranking within the top 2 for 7 of 9 times) (Supplementary Table S4; Supplementary Figure S3). Therefore, LD-pruned (*r*^2^ < 0.5) synonymous SNPs were used to construct GS models for each trait in each cohort. In total, we built 2094 GS models for 349 agronomically important traits, utilizing six different GS models including five traditional statistical models (GBLUP, rrBLUP, BayesCpi, BayesL and BayesR) and one machine-learning approach (LightGBM).

#### Development of SNPGT and CropGS-Hub reporting system

To enable users to utilize the constructed models for predicting traits of their own samples, we developed a user-friendly online platform within CropGS-Hub. However, building an online GS platform from sequencing reads (in FASTQ format) to predicted phenotypes can encounter various obstacles, such as efficient data uploading and genotyping. To address these challenges, especially for crops like maize with large genomes, we developed a desktop version of the SNP Genotyping Tool (SNPGT) compatible with both Linux and Windows systems. With this software, users without much command-line experience can handle genotyping for samples with thousands of SNP sites required by CropGS-Hub. Additionally, we implemented an *in silico* target site genotyping approach (see methods) to enhance genotyping efficiency (Supplementary Figure S1). We selected the rice cohort (GSTP008) containing filtered 6215 synonymous SNPs for GS prediction as a case to show the efficiency of SNPGT. This cohort comprises a diverse collection of cultivated rice varieties, with an extensive repertoire of agronomic traits, totaling to 51 traits. Therefore, we feel this cohort could be a very useful one for rice breeders to perform phenotypic prediction of their own samples. It was observed that the SNP genotyping process with this method was 10 times faster (12.97–20.07 times) compared to conventional genotyping, when the sequencing depth was above 5 × (5 – 20×). The performance efficiency becomes more obvious as sequencing depth increases. Additionally, the genotyping consistency was high, ranging from 99.98% to 99.68% (Supplementary Figure S1). Users simply need to upload the genotype file of their samples with thousands of SNP markers to CropGS-Hub. Moreover, our platform includes ‘User Model Training’ allowing crop scientists to generate models based on phenotypes and genotypes of their own populations.

Notably, a user-friendly reporting file in HTML format can be generated. This file lists the predicted trait values (based on all six models) for multiple samples in a dynamic table that allows for value sorting. A distribution plot of the target trait for the cohort is provided, aiding in the determination of sample percentiles for users. Moreover, the lead SNPs for GWAS significant signals of this trait based on our CropGS-Hub GWAS database are also listed in the report for users who are interested in traditional marker-assisted selection (MAS).

### CropGS-Hub functional modules Description

**Figure 3.**
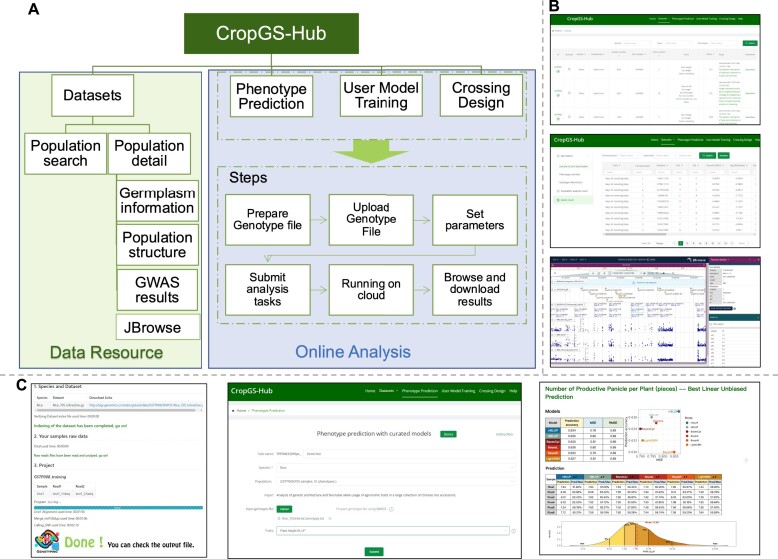
Overview of main modules and interfaces in CropGS-Hub. (**A**) Schematic diagram of data resource and online analysis in CropGS-Hub. (**B**) Interfaces of data resources. From up to bottom: interface for the information for each cohort, summary of GWAS associations, visualization of GWAS signals in JBrowse. (**C**) Examples of GS functional modules. From left to right: interface of SNPGT (windows version), job submission interface, the html-format result reporting interface.

CropGS-Hub is a comprehensive database that integrates meticulously curated genotype and phenotype resources. It also serves as a one-stop platform incorporating algorithms for genomic prediction and crossing design. The main functional modules of CropGS-Hub can be classified into two categories: genomic data resources and online genomic prediction (Figure 3A).

The website's navigation menu is divided into six sections: ‘Home’, ‘Datasets’, ‘Phenotype prediction’, ‘User model training’, ‘Crossing design’ and ‘Help’. The ‘Home’ page not only includes the title and website introduction but also provides navigation options for the database of the seven involved crop species and the main functional modules of the website. By clicking on the crop images, users can access the ‘Cohort’ page and view relevant population summary statistics, such as the number of SNPs, traits, and significant associations, for each species. Moreover, users can directly navigate to their desired functional modules by clicking on the corresponding sections displayed in the module overview. On the ‘Help’ page, users can find instructions and guidelines for each section of the website, ensuring a seamless user experience.

The ‘Datasets’ module consists of two functions: a search and browse page (named ‘Cohort’) of identified GWAS signals and variants information in each cohort, as well as an integrated GWAS results in multiple cohorts in each species (named ‘GWAS compare’). On the search and summary page, users can query specific populations curated in the database and browse summary information. The table includes species, sample types (inbred or hybrid), sample numbers, SNP numbers, traits numbers, significant GWAS loci numbers, source references, and download links for sample information, sample genotypes, and phenotype files (Figure 3B). By clicking on the population's unique identifier (GSTP ID), users can access the detailed information page for that population. Clicking on ‘JBrowse’ allows users to navigate to the JBrowse page specific to the population, where they can explore SNP information, GWAS results, and gene distribution.

The detailed information page of each population is divided into three sections: ‘Germplasm’, ‘Population analysis result’ and ‘GWAS result’ (Figure 3B). These sections offer valuable insights into the population. The ‘Germplasm’ section provides sample source information, phenotype distribution, modeling of each phenotype, and SNP distribution, utilizing a combination of graphics and lists. The ‘Population analysis result’ section presents PCA analysis results, evolutionary tree construction, and structure analysis based on SNPs at four-fold degenerate (4DTv) sites. The ‘GWAS result’ section displays significant GWAS results for SNPs associated with various agronomic traits. Users can search for specific chromosomal positions to view significant trait associations, and they can also click the ‘JBrowse’ button to access the JBrowse page specific to the population for further exploration. The GWAS result table includes detailed variant annotations such as genomic location, variant effect, deleterious level, and information about the gene at the locus and its *Arabidopsis* homolog. It is worth noting that all images on these pages can be clicked on to enlarge and view in more detail. In the ‘GWAS-compare’ page under Datasets module, Users could view the GWAS signal statistics in each species and enter to the detail information of GWAS results integrated in each species by clicking the species name button.

We have implemented three modules, namely ‘Phenotype Prediction’, ‘User Model Training’, and ‘Crossing Design’, for online genomic selection (GS) analysis. These modules utilize well-curated training populations and validated algorithms (Figure 3B). The ‘Phenotype Prediction’ page enables users to upload their own sample genotypes and select the trait of interest for phenotype prediction. The ‘Crossing Design’ module is designed specifically for hybridization prediction based on three trained F_1_ populations for maize and rice. The ‘Crossing Design’ module is specifically designed for hybridization prediction, using three trained F_1_ populations for maize and rice. With this module, users can select the cohort and utilize the SNPGT toolkit for genotyping samples that will be used for *in silico* crossing. There are two options for crossing: pairing all samples or pairing one sample with the remaining ones. After uploading the parental genotypes, *in silico*-generated F_1_ genotypes are subjected to modeling for phenotype prediction. This functionality could provide valuable guidance for parent selection in hybrid breeding. On the ‘User Model Training’ page, users can train GS models on our cloud server by uploading specific cohorts with genotype matrices and traits. Furthermore, users can use their models based on training population to predict their test samples.

Performing online analysis using the three modules on CropGS-Hub follows a simple process: preparing the genotype file, uploading it, setting parameters, submitting the analysis task, running jobs on the cloud, and browsing the results. Once the task is completed on our cloud server, users can view and download the results through the track link provided in the email notification. Our report is generated in HTML format and includes tables showing predicted values under the six GS models, as well as intuitive graphs displaying the percentile of the average predicted trait values with the samples of the specific cohort as the background. Users can download example data of the three pages by clicking on the corresponding ‘Demo’ button. Additionally, users can find information about these three functionalities on the ‘Help’ page. Genotype files for individuals in the three GS module pages can be obtained using our developed ‘SNPGT’ local plugin. This plugin facilitates efficient genotyping of our designed target site SNPs from sequencing data in FASTQ format.

### Use cases

#### Visualization of a candidate locus associated with rice grain width

**Figure 4.**
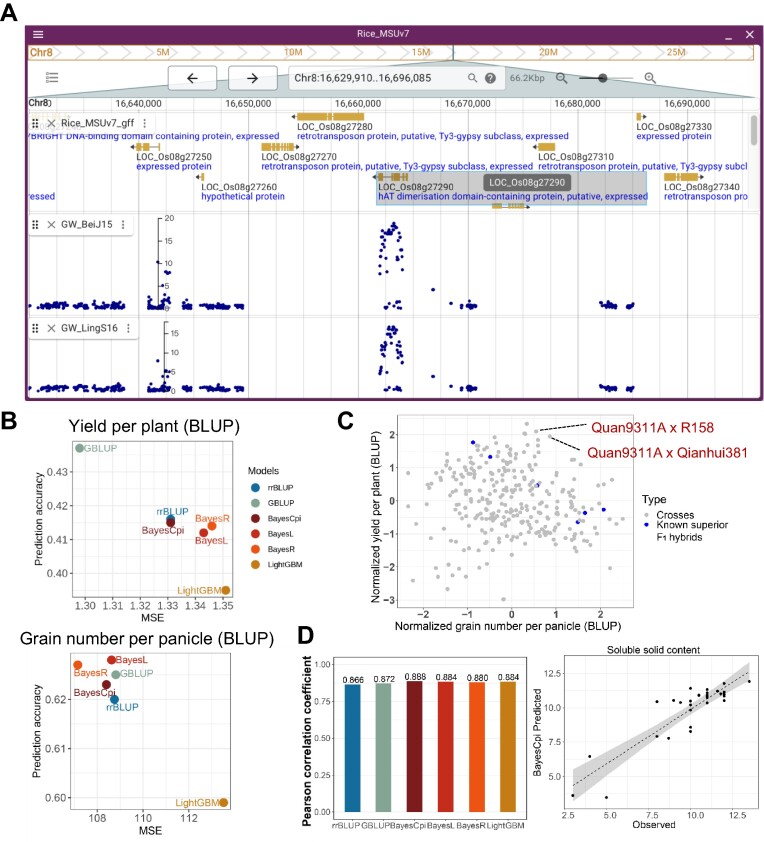
Use cases of CropGS-Hub. (**A**) JBrowse view displaying a significant GWAS signal associated with rice grain width. (**B**) Comparison of accuracy across different GS models for two yield-related traits of F_1_ hybrid rice in the ‘Crossing Design’ functional module. (**C**) Dot plot showing the predicted performance of 294 *in silico* F_1_ crosses with Quan9311A as the common parent. The X-axis represents the normalized number of grains per panicle, and the Y-axis represents the normalized yield per plant. The blue dots indicate previously developed superior *indica* crosses. (**D**) Utilizing the ‘User Model Training’ module of CropGS-Hub to predict the flesh sweetness of watermelon. Left: accuracy of six different models; Right: Pearson correlation between observed and predicted values of flesh sweetness level (measured by soluble solid content) using BayesCpi model for the test set comprising 30 samples.

Through scanning our GWAS database and gene-level association by MAGMA, we discovered a GWAS peak (Chr8: 16660959–16665094) associated with rice grain width. Notably, this narrow genomic region covers only one rice gene (LOC_Os08g27290) which is highly associated (*P* < 0.01) with the target trait by MAGMA analysis. Multiple SNPs within the gene showed significant associations, and the -log_10_(*P*) values for one lead SNP (8_16663267) in Beijing and Lingshui are 18.8 and 16.5, respectively (Figure 4A). According to SIFT (sorting intolerant from tolerant) evaluation, this SNP has been annotated as a deleterious variant with a SIFT score of 0.01. This gene has not been cloned in rice and thus a good candidate for further functional researches.

#### Prediction the potential of new rice crossing design

The commercial availability of 3-line hybrids dates back to the 1970s. One popular male sterile line, Quan9311A, has been successfully crossed with several restorer lines resulting in superior F_1_ hybrids such as ‘Quanyou727’, ‘Quanyou527’ and ‘Quanyouhuazhan’. To explore further possibilities, the ‘Crossing Design’ model of CropGS-Hub was used. Quan9311A was *in silico* crossed with 294 rice accessions commonly employed as restorer lines in 3-line rice hybrid breeding programs. The performance of ‘yield per plant’ and ‘grain number per panicle’ was predicted. Among the models used, GBLUP ranked as the most optimal model for ‘yield per plant’, while BayesL ranked highest for ‘grain number per panicle’ (Figure 4B). Analyzing the predicted performance of these 294 crosses, it was observed that the previously developed six superior F_1_ hybrids ranked in the top 30% for either of the two traits considered. Notably, CropGS-Hub also predicted several promising crosses with higher yield potential, such as the crosses involving ‘R158’, ‘Qianhui381’ and ‘R5128’ (Figure 4C).

#### Construction of GS models for the flesh sweetness of watermelon

The construction of CropGS-Hub not only facilitates genomic prediction for staple crops like rice and maize but also offers a feasible approach called ‘User Model Training’ for constructing and predicting GS models for any crops and animals. In this case, we focused on watermelon and constructed six different GS models to predict its flesh sweetness. The measurement of flesh sweetness was based on the soluble solid content, as described in Guo *et al.* (41). A dataset of 259 accessions was used, which included both the trait data and genotypes. Among these accessions, 229 were used for model training with 10-fold cross-validation, while the remaining 30 samples were used as a test population. We found that, for the test population, there was a high correlation (0.880–0.888) between the predicted flesh sweetness and the observed values across all the six GS models. The BayesCpi model showed the highest accuracy (0.888) among the constructed models (Figure 4D).

## Discussion

CropGS-Hub is the result of extensive collaboration among scientists in the molecular biology and genetics research communities. Our mission is to promote free access and efficient use of the rapidly expanding accumulation of population genomics data in the plant kingdom. In this study, we developed CropGS-Hub, a comprehensive platform that incorporates genotype and phenotype resources from 14 high-quality cohorts spanning 7 important crops. Leveraging these resources, we created a one-stop online genomic selection platform that integrates traditional statistics and machine learning models. To our knowledge, CropGS-Hub is a pioneering crop genome selection platform for multiple key crops, uniquely positioned to assist breeders in refining crop improvement strategies.

In addition to collecting high-quality genome and phenome data, CropGS-Hub conducts rigorous data cleaning and quality control procedures. This clean data is then used for germplasm distribution statistics, population analysis, and GWAS analysis. Additionally, we utilize JBrowse, an interactive tool, to visualize genotype data and GWAS results, allowing users to explore and understand the data more effectively. Our online services go beyond just connecting genomes and phenomes through GWAS. With our GS prediction modules, we establish a more accurate connection between genotype data and phenotypic values. We also provide phenotypic prediction, crossing design, and construction of GS models based on users’ data, each of which generates user-friendly html-format reports.

While current GS approaches and our CropGS-Hub platform have their strengths, they also have limitations. One significant limitation of traditional GS research is the heavy reliance on single linear genomes and SNP markers, potentially overlooking important structural variations (SVs) associated with traits not in linkage disequilibrium with adjacent SNPs. To address this, we propose adopting a ‘graph pan-genome’ approach that integrates extensive sequence and variation data from diverse populations. This comprehensive approach, as demonstrated by Zhou *et al.* (42) and Liao *et al.* (43), has the potential to improve the detection of complex variations, enhance genotyping accuracy, and recover ‘missing heritability’, thus increasing the accuracy and reliability of GS predictions. Incorporating this feature is a potential upgrade for future versions of CropGS-Hub.

Breeders involved in breeding programs aim to select plant materials that possess not only high yield but also good quality and strong disease resistance. One commonly used approach to consider multiple traits is the selection index (SI) method (44,45). With the selection index, each trait is assigned a weight, and an integrated score is generated based on a weighted linear combination of these traits. The weights are typically determined based on the economic importance of each trait and the breeders’ experiences regarding the optimal value range for each trait. Furthermore, in recent years, there has been a rise in the construction of multi-trait genome selection models (MTGS) that enable the simultaneous prediction of multiple traits. These MTGS models have shown potential in improving the predictability of traits with low heritability (46). Therefore, in future, we aim to enhance our platform by incorporating selection index and MTGS models, allowing users to predict and assess multiple traits simultaneously.

While GS frameworks excel in predictive capabilities and quantitative trait prediction, they often lack specific genetic guidance for breeders seeking to improve crops. By integrating quantitative trait nucleotides QTN-based ‘genetic testing’ approaches like RiceNavi (7), breeders can address the limitations of certain traits in seemingly promising materials. For example, if a rice variety exhibits favorable overall characteristics but lacks a specific trait predicted by CropGS-Hub, the RiceNavi approach can be employed to scan functional QTNs and identify loci that require genetic modifications. This integration could allow breeders to pinpoint specific genetic regions for targeted improvements, streamlining the breeding process and enhancing the overall effectiveness of GS-driven crop improvement.

## Supplementary Material

gkad1062_Supplemental_Files

## Data Availability

CropGS-Hub is available in https://iagr.genomics.cn/CropGS/. The SNPGT toolkit can be downloaded from https://iagr.genomics.cn/CropGS/#/snpgt.
