# Supplementary material for: CropGS-Hub: a comprehensive database of genotype and phenotype resources for genomic prediction in major crops
Source: Nucleic Acids Res. 2023 Nov 24;52(D1):D1519–29. doi: 10.1093/nar/gkad1062 (PMC10767954; doi:10.1093/nar/gkad1062)
Supplement: gkad1062_Supplemental_Files [file gkad1062_supplemental_files.zip › CropGS_SupplementaryFigures_1014.pdf]

Supplementary Figure S1

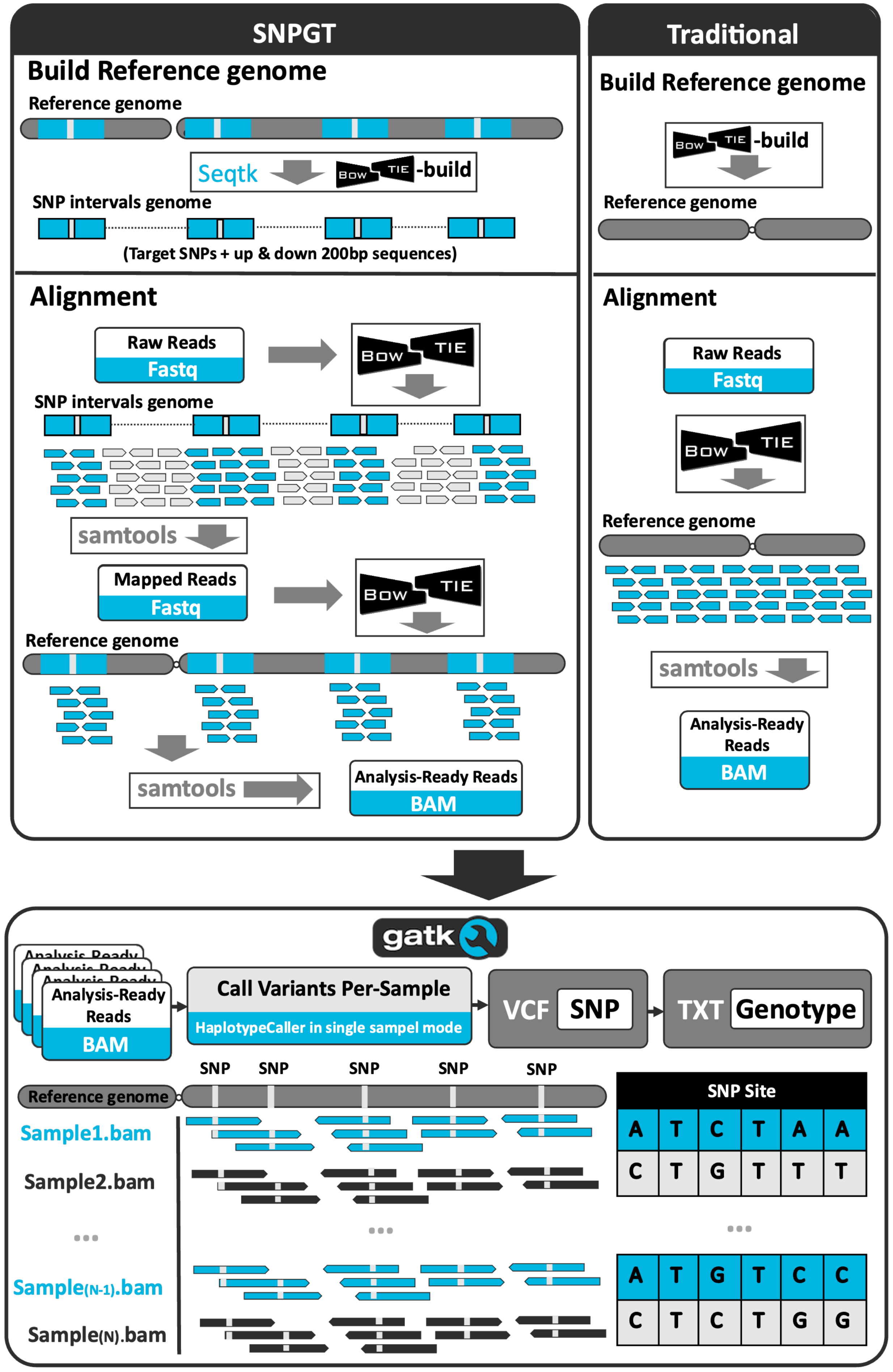

Depth	SNPGT Step	Time	Traditional Step	Time (s)	Time ratio (Trad/SNPGT)
1 Fold	Alignment SNP intervals	8.29	Alignment Reference genome	36.97	2.42
	bam -> fastq	3.93			
	Alignment Reference genome	3.08			
	Sort bam	5.17	Sort bam	161.85	31.31
	Removal of PCR duplication	5.64	Removal of PCR duplication	180.02	31.92
	Call Variants Per-Sample	103.81	Call Variants Per-Sample	333.14	3.21
	**All steps**	**129.92**	**All steps**	**711.98**	**5.48**
2 Fold	Alignment SNP intervals	16.21	Alignment Reference genome	66.71	2.32
	bam -> fastq	7.58			
	Alignment Reference genome	5.01			
	Sort bam	9.61	Sort bam	327.57	34.09
	Removal of PCR duplication	10.67	Removal of PCR duplication	350.09	32.81
	Call Variants Per-Sample	102.03	Call Variants Per-Sample	627.41	6.15
	**All steps**	**151.11**	**All steps**	**1371.78**	**9.08**
5 Fold	Alignment SNP intervals	44.29	Alignment Reference genome	142.78	1.84
	bam -> fastq	20.41			
	Alignment Reference genome	12.77			
	Sort bam	23.98	Sort bam	816.89	34.07
	Removal of PCR duplication	25.31	Removal of PCR duplication	827.69	32.70
	Call Variants Per-Sample	129.17	Call Variants Per-Sample	1532.81	11.87
	**All steps**	**255.93**	**All steps**	**3320.17**	**12.97**
10 Fold	Alignment SNP intervals	64.05	Alignment Reference genome	275.65	2.27
	bam -> fastq	37.14			
	Alignment Reference genome	19.99			
	Sort bam	47.31	Sort bam	1604.47	33.91
	Removal of PCR duplication	48.03	Removal of PCR duplication	1592.23	33.15
	Call Variants Per-Sample	164.14	Call Variants Per-Sample	3436.89	20.94
	**All steps**	**380.66**	**All steps**	**6909.24**	**18.15**
15 Fold	Alignment SNP intervals	102.40	Alignment Reference genome	418.36	2.24
	bam -> fastq	53.43			
	Alignment Reference genome	31.10			
	Sort bam	71.55	Sort bam	2507.69	35.05
	Removal of PCR duplication	69.47	Removal of PCR duplication	2369.10	34.10
	Call Variants Per-Sample	227.05	Call Variants Per-Sample	5079.21	22.37
	**All steps**	**555.00**	**All steps**	**10374.36**	**18.69**
20 Fold	Alignment SNP intervals	122.31	Alignment Reference genome	548.39	2.35
	bam -> fastq	73.21			
	Alignment Reference genome	38.12			
	Sort bam	96.14	Sort bam	3231.57	33.61
	Removal of PCR duplication	90.15	Removal of PCR duplication	3103.39	34.42
	Call Variants Per-Sample	270.30	Call Variants Per-Sample	6971.10	25.79
	**All steps**	**690.23**	**All steps**	**13854.45**	**20.07**

Depth	Marker number	Difference between SNP and traditional approach	Consistency rate
1 Fold	6215	0	100.00%
2 Fold		1	99.98%
5 Fold		1	99.98%
10 Fold		9	99.86%
15 Fold		12	99.81%
20 Fold		20	99.68%

Supplementary Figure S1. Design of SNPGT pipeline and comparison of efficiency between SNPGT and conventional genotyping approach.

Supplementary Figure S2

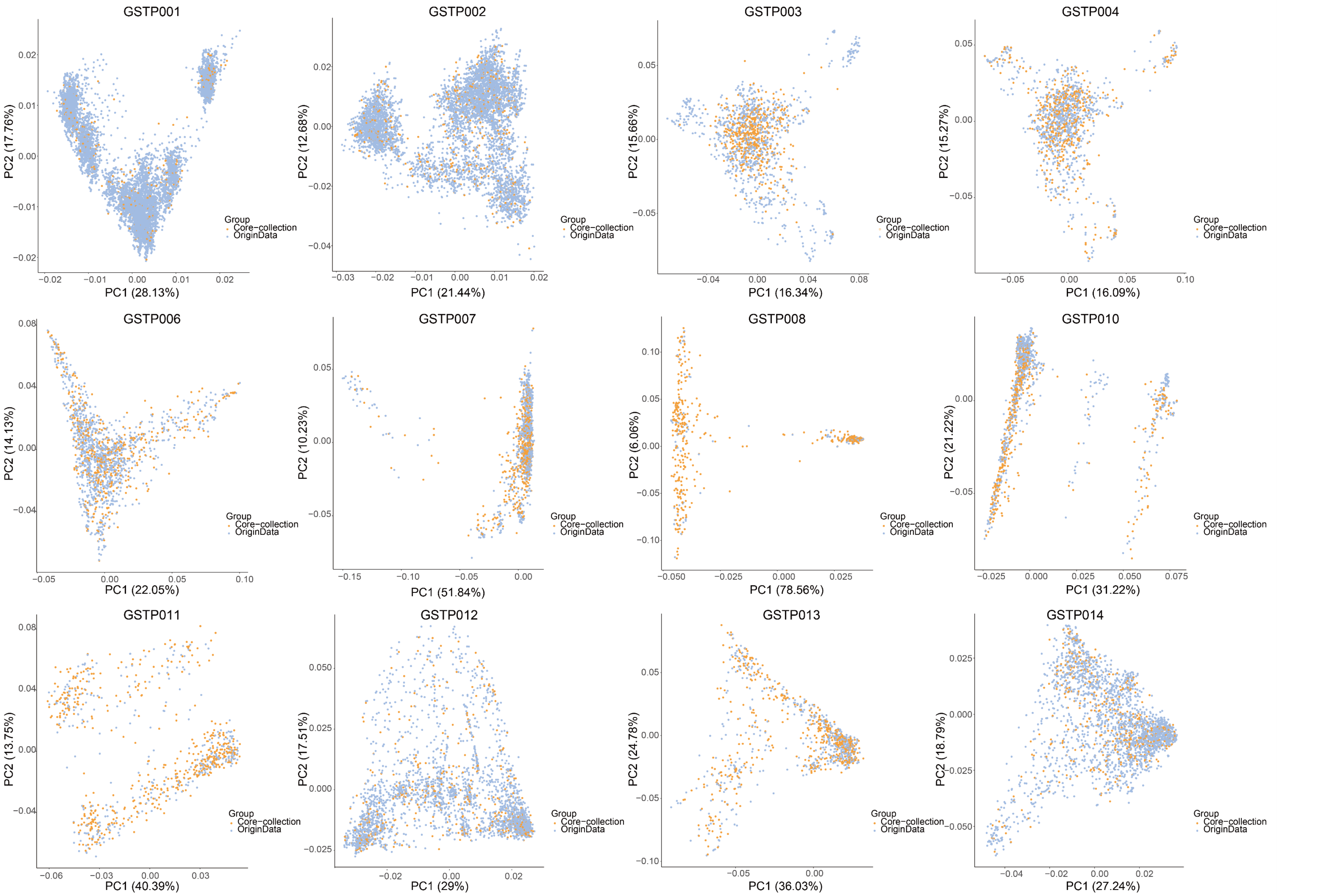

Supplementary Figure S2. PCA plot of 12 cohorts with mini-core collection whose samples exceeded 500. The dot colors correspond to the origin samples and mini-core collection samples.

Supplementary Figure S3

(A)

Species	Trait	Intergenic	Intronic	Tolerated	Deleterious	Synonymous
Maize	Plant height	0.5960	0.5951	0.6138	0.6123	0.6174
	Grain yield per plant	0.6646	0.6739	0.6712	0.6650	0.6734
	Days to anthesis	0.7374	0.7451	0.7490	0.7523	0.7601
Rice	Plant height	0.7649	0.7661	0.7566	0.7572	0.7641
	Grain yield per plant	0.5726	0.5685	0.5726	0.5642	0.5778
	Heading date	0.8993	0.8984	0.8942	0.8964	0.8981
Soybean	Plant height	0.7966	0.8069	0.8088	0.7969	0.8092
	One Hundred seed weight	0.7821	0.7813	0.7735	0.7733	0.7822
	Beginning bloom date	0.8465	0.8486	0.8514	0.8456	0.8492

(B)

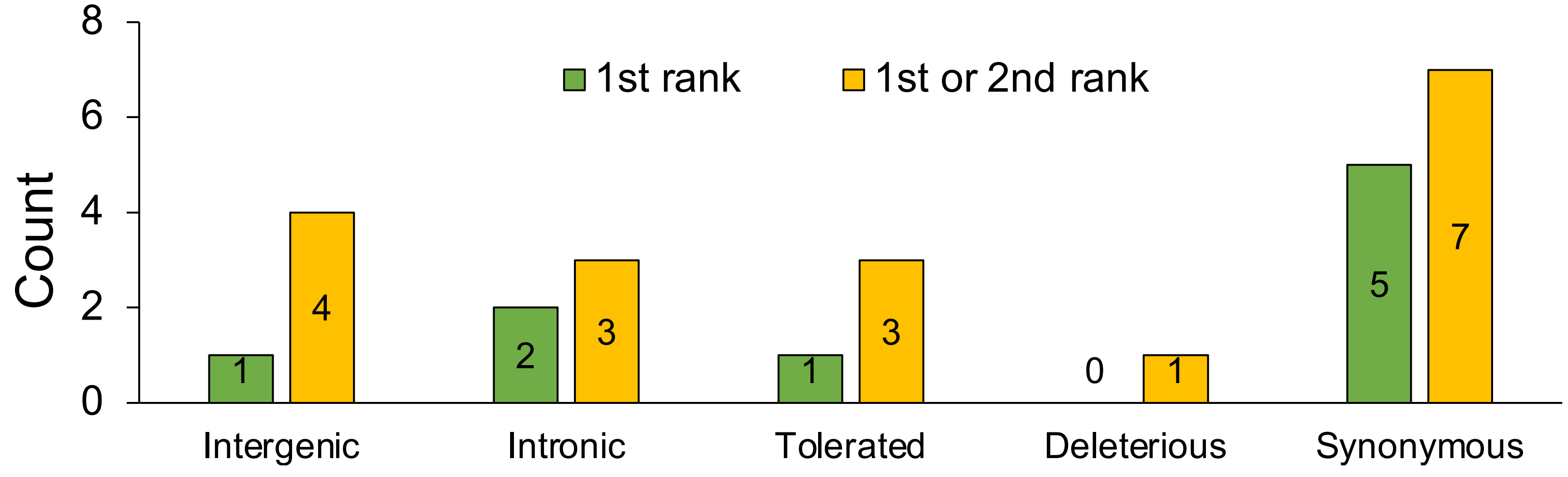

Supplementary Figure S3. Benchmarking the prediction accuracy based on different SNP types. (A) The average accuracy of predictions based on five GS models (provided in Supplementary Table 4) for three traits of each three species. (B) The frequency at which each SNP type ranks within the top 2 is calculated.
